# ACBP/DBI neutralization for the prevention and treatment of malignant and non-malignant liver diseases

**DOI:** 10.1038/s41419-025-08148-0

**Published:** 2025-11-28

**Authors:** Sijing Li, Flavia Lambertucci, Léa Montégut, Isabelle Martins, Jonathan Pol, Mauro Piacentini, Maria Chiara Maiuri, Guido Kroemer

**Affiliations:** 1 Team « Metabolism, Cancer & Immunity », Équipe labellisée par la Ligue contre le cancer, Centre de Recherche des Cordeliers U1138, Inserm, Université Paris Cité, Sorbonne Université, Paris, France; 2 Department of Epidemiology, Preclinical Research and Advanced Diagnostics, National Institute for Infectious Diseases IRCCS ‘L. Spallanzani’, Rome, Italy; 3 National Institute for Infectious Disease IRCCS “Lazzaro Spallazani”, Rome, Italy; 4 Department of Molecular Medicine and Medical Biotechnologies, University of Napoli Federico II, Naples, Italy; 5 Université Paris-Saclay, INSERM US23/CNRS UAR 3655, Metabolomics and Cell Biology Platforms, Institut Gustave Roussy, Villejuif, France; 6 Institut du Cancer Paris CARPEM, Department of Biology, Hôpital Européen Georges Pompidou, AP-HP, Paris, France

**Keywords:** Liver cancer, Liver cancer

## Abstract

Acyl coenzyme A binding protein (ACBP), also known as diazepam binding inhibitor (DBI), suppresses autophagy, stimulates food intake, and regulates body composition. This tissue hormone contributes to the development of age-related diseases such as metabolic syndrome, cardiovascular disease, cancer, and osteoarthritis. ACBP/DBI also plays a key pathogenic role in liver disorders, including hepatocellular carcinoma (HCC). Circulating levels of ACBP/DBI are elevated in patients with histologically diagnosed steatosis, liver fibrosis or HCC, and correlate with disease severity. Moreover, the incidence of liver cancers increases in individuals receiving benzodiazepines, which act on the same binding sites of the GABA-A receptor as ACBP/DBI. In mice, inhibiting ACBP/DBI, via inducible knockout, mutation of its receptor (the γ2 subunit of the GABA-A receptor) or antibody-mediated neutralization, alleviates various liver conditions, including ischemia-reperfusion injury, bile duct obstruction, hepatotoxicity of acetaminophen, CCl_4_, ethanol, or concanavalin A, metabolic dysfunction-associated fatty liver disease, and HCC. Importantly, the anti-tumor effects of ACBP/DBI neutralization are not solely due to its hepatoprotective properties, as they persist in mouse models of HCC driven by oncogenes (e.g., β-catenin and MYC) or orthotopic injection of syngeneic liver cancer cells into immunocompetent hosts. Notably, hepatocellular carcinoma (HCC) is one of the few cancers in which elevated local ACBP/DBI expression is associated with poor clinical prognosis. In sum, ACBP/DBI functions as both a biomarker and a potential therapeutic target for malignant and non-malignant liver diseases.

## Facts

- Source of circulating ACBP/DBI in liver disease: Plasma ACBP/DBI levels are elevated in fatty liver disease, fibrosis, and hepatocellular carcinoma. However, the relative contributions of normal versus transformed hepatocytes to this increase remain unclear.
- Intracellular vs. extracellular roles: Intracellular ACBP/DBI supports lipid metabolism and cell survival, whereas extracellular ACBP/DBI suppresses autophagy. While neutralizing extracellular ACBP/DBI exerts strong hepatoprotective and cancer-preventive effects, it is not yet known whether targeting intracellular ACBP/DBI could provide additional therapeutic benefits.
- Generalizability: In hepatocellular carcinoma, ACBP/DBI promotes immune evasion and inhibits ferroptosis. Evidence from breast, lung, and skin cancers indicates that ACBP/DBI broadly suppresses anticancer immunity. Whether its role in ferroptosis extends to cancers beyond the liver remains to be determined.
- Translation to humans: In mice, ACBP/DBI neutralization has demonstrated both prophylactic and therapeutic benefits. The efficacy of ACBP/DBI-neutralizing antibodies in protecting the liver and suppressing tumors in humans still requires clinical validation.

## Introduction

The liver can be viewed as the brain of metabolism—a central command hub that constantly receives information on the levels of systemic or gut-derived nutrients, hormones, and neuroendocrine signals [1, 2]. Indeed, hepatocytes, which compose the parenchyma of this major organ, are unique in that they interface directly with the lymphatic system and both systemic and portal (gut-derived) blood circulation. Like a seasoned strategist processing intelligence from every corner of the body, the liver decodes these messages and formulates precise metabolic responses. Its decisions ripple outward, influencing the behavior of virtually every organ—dictating energy supply, affecting nutrient absorption, calibrating biosynthesis, orchestrating detoxification, and modulating immune activity [3, 4]. Just as the brain governs thought and action through a web of neuronal circuits, the liver oversees physiological balance through an intricate metabolic and endocrine dialog with the entire organism [5]. Hepatocytes metabolize carbohydrates, proteins, and lipids, maintaining energy balance through glycogenesis, gluconeogenesis, and β-oxidation. They also synthesize plasma proteins like albumin and clotting factors, and detoxify xenobiotics and endogenous waste such as ammonia via the urea cycle. Additionally, the liver regulates cholesterol levels, produces bile for digestion and fat absorption, and stores essential vitamins, iron and copper.

The liver is susceptible to a spectrum of pathologies characterized by progressive disruption of hepatocellular function, chronic inflammation, and architectural remodeling. A major category is metabolic dysfunction-associated steatotic liver disease (MASLD), formerly distinguished as non-alcoholic fatty liver disease (NAFLD), encompassing a continuum from simple steatosis to metabolic dysfunction-associated steatohepatitis (MASH) and terminal cirrhosis [6]. MASLD is defined by hepatic steatosis in the context of metabolic risk factors, such as obesity, insulin resistance, and dyslipidemia, and is associated with lipotoxicity, mitochondrial dysfunction, and sterile inflammation, ultimately promoting fibrosis. Alcohol-related liver disease (ALD), now conceptualized within the broader umbrella of steatotic liver disease, shares overlapping pathomechanisms, including oxidative stress, impaired autophagy, and perturbations of the gut-liver axis [6]. Across etiologies, fibrogenesis, driven by activated hepatic stellate cells and extracellular matrix deposition, is a unifying mechanism leading to cirrhosis and an elevated risk of hepatocellular cancer (HCC) [7]. However, any kind of chronic liver inflammation including MASH without manifest cirrhosis can lead to HCC. For this reason, metabolic dysfunction-associated HCC is on the verge of becoming more frequent than liver cancer caused by chronic viral hepatitis [8, 9].

Macroautophagy is a fundamental catabolic process that maintains hepatocyte function by sequestering damaged organelles, misfolded proteins, and excessive lipid droplets in autophagosomes that then fuse with lysosomes for digestion of the luminal content by hydrolases operating at an acidic pH [10]. In liver cells, macroautophagy mobilizes intracellular nutrient resources during fasting, facilitates lipid catabolism through lipophagy, and mitigates oxidative stress by selectively destroying dysfunctional mitochondria (mitophagy). This dynamic turnover is critical for suppressing chronic inflammation, endoplasmic reticulum (ER) stress, and oncogenic transformation [10]. Reduction of autophagic flux has been implicated in the pathogenesis of MASLD, ALD, and HCC. In oncogenic contexts, impaired autophagy contributes to hepatocarcinogenesis through multiple converging mechanisms: defective mitophagy leads to accumulation of reactive oxygen species (ROS) and oxidative DNA damage; impaired protein quality control promotes aggregation of oncogenic proteins; and unresolved ER stress activates pro-tumorigenic signaling pathways such as JNK and NF-κB. Moreover, reduced autophagy limits the turnover of p62/SQSTM1, a selective autophagy receptor whose accumulation can activate the KEAP1–NRF2 axis, spurring unwarranted cell survival and proliferation [11, 12]. Indeed, hepatocyte-specific deletion of core autophagy genes such as *Atg5* or *Atg7* is sufficient to initiate hepatocarcinogenesis in mouse models, underscoring the tumor-suppressive role of basal autophagy in liver tissue [13].

Acyl-CoA binding protein (ACBP), also known as diazepam-binding inhibitor (DBI), is a small, evolutionarily conserved protein that binds intracellular lipids, including medium- and long-chain acyl-CoA esters, thereby regulating their metabolism in a wide range of cell types [14–17]. Thus, intracellular ACBP/DBI facilitates the metabolism of unsaturated fatty acids in astrocytes [18], favors fatty acid metabolism in glioblastoma [19] and bone metastases of breast and lung cancer, [20] represses adaptive thermogenesis in brown adipose tissue, [21] and favors maladaptive cardiac remodeling in the context of diabetes [22], as demonstrated in tissue-specific conditional knockout experiments. Beyond its cell-autonomous metabolic role, ACBP/DBI functions as an extracellular signaling molecule that is secreted in response to autophagy induction or passively released in the context of cell death. Once in the extracellular space, ACBP/DBI acts through paracrine or autocrine mechanisms, engaging γ-aminobutyric acid (GABA) type A receptors to modulate cell fate and inflammation [23]. Intriguingly, ACBP/DBI appears to form part of a feedback loop that limits autophagic activity: while autophagy promotes its secretion, extracellular ACBP/DBI in turn suppresses autophagy, thus serving as a brake on prolonged catabolic activation or “autophagy checkpoint” [16, 24]. Neutralization of extracellular ACBP/DBI—using antibodies, genetic ablation or receptor mutation—has been shown to exert potent cytoprotective, anti-inflammatory, anti-fibrotic, and tumor-suppressive effects in multiple preclinical models, including in several paradigms of liver disease [23–28]. These beneficial outcomes are at least in part attributable to the restoration of autophagic flux and the attenuation of pro-inflammatory signaling, positioning ACBP/DBI as a novel regulator at the interface of lipid metabolism, autophagy, and tissue homeostasis. Indeed, the effects of ACBP/DBI inhibition on healthspan are that pronounced [16, 29], that ACBP/DBI has been classified as a pro-aging gene (product) or “gerogene” [30].

Here, we will discuss current evidence suggesting that the ACBP/DBI system can be targeted for the prevention or treatment of a wide range of liver diseases.

## Evidence implying ACBP/DBI in human liver diseases

Plasma ACBP/DBI concentrations can be conveniently measured by means of ELISA and proteomics technologies (including the latest versions of the Olink and Somascan technologies) [31–33]. ACBP/DBI plasma concentrations increase with age and with body-mass index (BMI), as observed in several cohorts of apparently healthy adult individuals [31, 32, 34, 35]. In addition, plasma ACBP/DBI correlates with multiple parameters indicative of metabolic syndrome such as arterial hypertension, glycemia, free fatty acids, total cholesterol, low-density lipoprotein (LDL), and reduced levels of high-density lipoprotein (HDL) [35]. In accord with the fact that advanced age, elevated BMI and metabolic syndrome are risk factors for a range of liver diseases, plasma ACBP/DBI concentration also correlate with biochemical parameters indicative of liver damage, such as elevations of circulating alanine aminotransferase, alkaline phosphatase, and γ-glutamyltransferase (Table 1). In addition, in a mixed population of individuals that were either normal or diagnosed with NAFLD, ACBP/DBI plasma levels correlate with signs of reduced liver function, such as an increase in plasma bilirubin or a decrease in albumin levels (Table 1). ACBP/DBI plasma concentrations also correlate with composite scores that measure the severity of liver disease [27], as this applies to the NAFLD fibrosis score, which integrates four blood parameters (albumin, alanine transaminase [ALT], aspartate transaminase [AST], and platelet count) and three clinical parameters (age, body mass index [BMI], diabetes) [36], as well as to the FIB4 score, which only computes four parameters (age, ALT, AST, and platelet count) [37].

**Table 1:** Liver diseases correlating with elevated ACBP/DBI plasma levels.

Disease category	Parameter or disease	Reference
Non-malignant	Alanine aminotransferase (ALT) in plasma	[27, 31]
	Alkaline phosphatase in plasma	[27, 31, 32]
	γ-glutamyltransferase in plasma	[31, 32]
	Bilirubin in plasma	[27, 35]
	Reduced albumin in plasma	[27, 35]
	NAFLD score	[27]
	FIB4 score	[27]
	Histologically diagnosed MASH (categoric variable)	[27]
	Histologically diagnosed liver fibrosis (categoric variable)	[27]
	Histologically diagnosed cirrhosis (categoric variable)	[38]
Malignant	α-fetoprotein in plasma	[25]
	Largest liver tumor size	[25]
	Histologically diagnosed hepatocellular carcinoma compared to controls (categoric variable)	[25]
	Advanced BCLC stage (B/C > O/A, categoric variable)	[25]
	Vascular invasion (categoric variable)	[25]
	Metastasis (categoric variable)	[25]

More importantly, ACBP/DBI plasma concentrations turned out to be higher in patients that had undergone liver biopsies with histological evidence of steatosis, fibrosis or cirrhosis as compared to controls without any signs of liver pathology [27, 38]. This finding extends to histologically diagnosed HCC, in which ACBP/DBI plasma concentrations are higher than in tumor-free controls, in particular in patients with poor prognosis features such as advanced BCLC stages, vascular invasion and extrahepatic metastases [25]. Importantly, ACBP/DBI plasma levels correlated with those of circulating α-fetoprotein, as well as the radiologically determined largest liver tumor size [25] (Table 1).

**Fig. 1:**
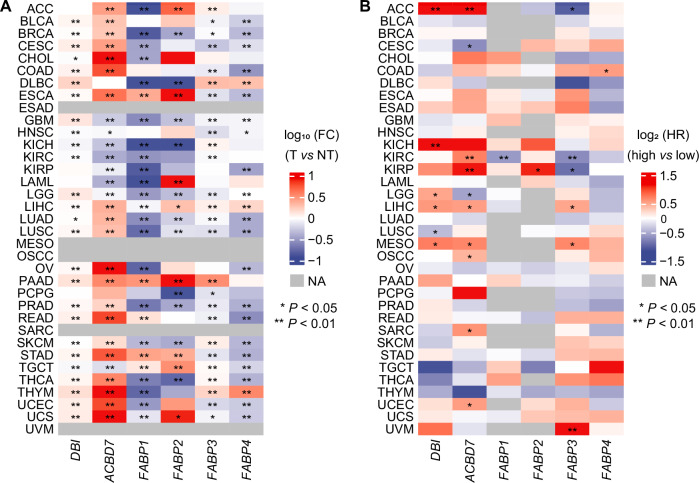
Impact of local mRNA expression of various genes coding for fatty acid binding proteins on patient prognosis across different cancers in the TCGA database. **A** Differential gene expression across cancer types. Fold change (FC) was calculated based on mean gene expression (log_2_ (TPM + 1)) in tumor (T) versus non-tumor (NT). The log_10_-transformed FC is presented in the heatmap. *P* values were calculated by means of the Wilcoxon rank sum test. **B** Kaplan–Meier survival analyses of gene mRNA expression across distinct cancers. Overall survival (OS) was calculated using the log-rank test, with patients stratified into high and low expression groups based on the median gene expression level (log₂ (TPM + 1)) for each cancer type. The log₂-transformed hazard ratio (HR) is presented in the heatmap. *P < 0.05, **P < 0.01. The data were obtained from the UCSC Xena platform (https://xenabrowser.net/datapages/), incorporating tumor samples from The Cancer Genome Atlas (TCGA) and non-tumor samples from the Genotype-Tissue Expression (GTEx) project.

The question then comes up whether the diseased liver itself is the source of circulating ACBP/DBI. The secreted liver proteome measured in the supernatant of precision-cut slides from liver biopsies that were cultured for 16 h contained higher ACBP/DBI concentrations in MASH than in non-pathological controls as well as in non-MASH MASLD controls [39], supporting the idea that the liver from MASH patients produces high levels of ACBP/DBI. When human HCC cells were orthotopically inoculated into the livers from immunodeficient mice, the levels of human ACBP/DBI (but not those of mouse ACBP/DBI) detectable in the plasma correlated with the size of the tumors, suggesting that HCC itself might constitute a source for circulating ACBP/DBI [25]. Accordingly, there is abundant evidence that local expression of ACBP/DBI mRNA is elevated in human liver cancers compared to normal adjacent tissue. This applies to 10 RNAseq studies comparing gene expression in HCC with normal liver tissues [25]. In HCC patients, local ACBP/DBI mRNA levels correlate with poor overall survival. Although ACBP/DBI is upregulated at the transcriptional level in multiple different human cancer types (Fig. 1A), this ACBP/DBI mRNA expression only correlates with poor overall survival in a selected fraction of cancer types, including HCC, several kidney cancer subtypes, and two rather rare tumor entities, namely, adrenocortical carcinoma, low grade glioma and mesothelioma) (Fig. 1B). Of note, in HCC, the closest human homolog of ACBP/DBI, ACBD7, although less abundantly expressed in peripheral tissues, was also both upregulated and associated with poor prognosis. In contrast none of the fatty acid biding proteins (FABP1, 2, 3 and 4) demonstrated a coherent pattern of upregulation and poor prognostic significance in HCC (Fig. 1).

In sum, both malignant and non-malignant human liver pathologies are associated with an elevation of circulating ACBP/DBI that likely stems from the diseased organ. In addition, HCC is coupled to the upregulation of ACBP/DBI mRNA levels, correlating with dismal prognosis.

## Benzodiazepine use increases the risk of liver cancer in population studies

ACBP/DBI is also called “endozepine” because it acts as an endogenous benzodiazepine equivalent [17]. Indeed, ACBP/DBI binds to exactly the same sites in the GABRG2 subunit of GABAAR as does the prototypic benzodiazepine diazepam. Hence, the prolonged use of benzodiazepines may be expected to have the same consequences as a chronic overstimulation of the ACBP/DBI system.

Importantly, in human population studies, the use of benzodiazepines has been linked to an increase in the incidence of liver cancers, as this has first been shown in a population-based retrospective analysis of a large Taiwanese study matching close to 60,000 benzodiazepine users with controls by age and sex. This study found an increase in liver cancer risk (hazard ratio = 1.45; 95% confidence interval 1.10–1.90) [40]. Similarly, in the Danish nationwide registry, close-to 150,000 cases with a first-time cancer who were matched (1:8) by age and gender to 1,200,000 cancer-free controls. In this study, long term use of benzodiazepines and related drugs (BZDR) was defined by a cumulative amount of ≥500 defined daily doses of BZRD within a period of 1 to 5 years prior to the index date. The adjusted odds ratio (OR) of long-term BZRD use for liver cancer was highly significant (odds ratio = 1.81, 95% confidence interval 1.18–2.80) compared to controls, and this effect remained significant after applying propensity score calibration to eliminate confounding factors [41, 42]. Finally, a meta-analysis of observational studies including a total of 18 case-control studies and 4 cohort studies (approximately 200,000 patients with cancer and 1.7 million controls) confirmed that benzodiazepine use was significantly associated with an increased risk of liver cancer (odds ratio = 1.22, 95% CI 1.13–1.31) [43].

Altogether, these population studies indicate that the use of benzodiazepines is associated with an increased likelihood of developing liver cancer, lending further indirect support for the implication of ACBP/DBI in hepatic carcinogenesis.

## Experimental methods to modulate the ACBP/DBI system in mice

The aforementioned findings obtained on human samples cannot inform on possible cause-effect relationships and hence suggest, but do not proof, a pathogenic role for ACBP/DBI in liver disease. To resolve this question, we resorted to preclinical models in mice, using an orthogonal approach to activate or inhibit the ACBP/DBI system (Table 2).

**Table 2:** Methods for manipulating the ACBP/DBI system in mice.

Purpose	Category	Method	Reference
Activation	Genetic	Transgenic overexpression of *Dbi* in liver	[24]
		Chemical-genetic system: Expression of a biotin-activatable transgene encoding secreted ACBP/DBI protein in hepatocytes	[45]
	Pharmacological	Intravenous injections of recombinant ACBP/DBI protein	[24]
		Subcutaneous osmotic pumps releasing ACBP/DBI or ACBP/DBI-derived neuropeptides (TTN or ODN)	[45]
		Intraperitoneal injection of diazepam for activation of the GABA A receptor	[56]
Inhibition	Genetic	Tamoxifen-inducible, body-wide knockout of *Dbi*	[24]
		Conditional knockout of *Dbi* in hepatocytes	[46, 49]
		Constitutive mutation F77I in *Gabrg2*, blocking interaction with ACBP/DBI	[23, 46]
	Immunological	Autoimmunization with ACBP/DBI-KLH conjugate	[24, 50]
		Monoclonal antibodies neutralizing ACBP/DBI injected i.v. or i.p.	[23, 24, 28]

For the stimulation of the ACBP/DBI system, we used two different transgenic approaches based on the hydrodynamic injection of plasmids that, due to the administration process, are preferentially expressed in hepatocytes. As a first approach, we injected plasmids encoding native ACBP/DBI (which is a leaderless peptide of 86 amino acids) into mice, causing the expression of ACBP/DBI in the liver, as well as systemic metabolic effects leading to an increase in food intake and body weight [24]. However, this approach was not able to distinguish between cell-autonomous effects of intracellular ACBP/DBI on hepatocytes and effects of extracellular ACBP/DBI inside and outside of the liver. Hence, as a second approach, we constructed a chemical genetic system based on the RUSH (retention using specific hooks) method [44]. For this, ACBP/DBI was fused in its N-terminus with a streptavidin-binding peptide (SBP) motif, separated by a linker containing a furin cleavage site [45]. The plasmid encoding the SBP-ACBP/DBI fusion protein was delivered by hydrodynamic injection together with another plasmid coding for streptavidin that, due to a KDEL motif, is sequestered in the lumen of the endoplasmic reticulum (ER). When provided in excess, this ER-located streptavidin locally retains the SBP-ACBP/DBI fusion protein. However, upon administration of biotin, which has a ~1000-fold higher affinity for streptavidin than SBP, the vitamin competitively disrupts the interaction between streptavidin and SBP-ACBP/DBI, allowing this latter chimeric protein to translocate into the Golgi, to be cleaved by the Golgi-sessile protease furin and then to be secreted through the conventional protein secretion pathway into the extracellular space [45]. In other words, intraperitoneal injection of biotin or its oral supplementation causes an increase in plasma ACBP/DBI levels due to its active secretion by transgenic hepatocytes co-expressing ER-sessile streptavidin and SBP-ACBP/DBI. This model has been used to show that extracellular ACBP/DBI can stimulate appetite and prevent stress-induced anorexia or chemotherapy-induced cachexia in suitable mouse models, hence unraveling a major role for ACBP/DBI in determining body composition [45].

A more direct, but also more stressful, method for stimulating the ACBP/DBI system consists in daily intravenous injections of recombinant ACBP/DBI protein or the subcutaneous implantation of osmotic pumps releasing either ACBP/DBI or neuropeptide derived thereof such as triakontatetraneuropeptide (TTN corresponding to amino acids 17 to 50) or octadecaneuropeptide (ODN corresponding to amino acids 33 to 50) [45]. Both the chemical-genetic system explained above and the periodic injection of recombinant ACBP/DBI protein lose their appetite-stimulatory effects in mice bearing a point mutation (F77I) in the gamma-2 subunit of the GABA A receptor (GABRG2) that abolishes binding of ACBP/DBI [23, 45, 46]. Hence, this mutation (mouse genotype: *Gabrg2*^F77I/F77I^) offers another possibility to constitutively block the extracellular action of ACBP/DBI.

Depending on how ACBP/DBI is knocked out in mice, the outcomes vary. Thus, upon constitutive knockout of the *Dbi* gene in the germline, embryonic lethality can occur as early as the morula stage [47]. Alternatively, constitutive knockout may lead to postnatal lethality around the weaning stage due to a keratinocyte differentiation defect [48]. For this reason, we resorted to an inducible knockout (by a ubiquitously expressed tamoxifen-inducible CRE recombinase) of exon-2-floxed *Dbi*, observing that the bodywide removal of ACBP/DBI at the adult stage (6–8 weeks) did not cause any deleterious effects and actually blunted excessive weight gain induced by high-fat diet [24]. Such adult-stage *Dbi* knockout mice were resistant against the induction of several malignant or non-malignant liver diseases (see below). Moreover, we performed the conditional knockout of ACBP/DBI in hepatocytes mediated by CRE recombinase expressed under the control of the albumin promoter [46, 49]. This organ-specific knockout has no obvious phenotype, yet is resistant to the induction of glucocorticoid-induced steatosis and oncogene-induced HCC (see below), hence providing yet another tool for investigating the pathogenic impact of ACBP/DBI (Table 2).

Beyond these genetic methods to remove both intracellular and extracellular ACBP/DBI, we used several immunological and pharmacological methods to inhibit ACBP/DBI. Extracellular ACBP/DBI can be intercepted by antibodies that are induced by autovaccination with adjuvantized ACBP/DBI (fused to keyhole limpet hemocyanin, KLH, for optimal immunogenicity) or by monoclonal antibodies that are injected either intraperitoneally or intravenously [23, 24, 28, 50]. Alternatively, extracellular ACBP/DBI can be functionally inhibited by means of an eicosapeptide derived from GABRG2 (flanking the critical F77 residue) that blocks the ACBP/DBI-GABRG2 interaction [51]. Finally, resmetirom, a selective thyroid hormone receptor beta agonist approved for the treatment of MASH, can be used to block the transcription of the *Dbi* gene in hepatocytes [49] (Table 2).

In sum, a whole arsenal of genetic, immunological and pharmacological methods allows to explore the ACBP/DBI system by its overactivation or inhibition. In our opinion, it is important to compare the pathophysiological consequences of distinct methods of manipulating the ACBP/DBI system for their orthogonal cross-validation.

## Experimental targeting of non-malignant liver diseases by inhibiting ACBP/DBI

The first hypothesis that ACBP/DBI might affect liver (patho)physiology was grounded in the observation that autoantibodies against ACBP/DBI reduced hepatosteatosis in mice fed a high-fat diet [24]. However, this effect on hepatosteatosis might have been secondary to reduced appetite and weight gain [24]. A second round of observations, however, suggested that this anti-steatotic effect of ACBP/DBI inhibition was not the consequence of reduced calorie intake. Indeed, ACBP/DBI neutralization had anti-MASH effects against a methionine/choline-deficient diet (MCD) that causes liver steatosis in the context of weight loss (rather than weight gain), both in a prophylactic setting (when anti-ACBP/DBI antibodies were administered before MCD) [23], as well as in a therapeutic setting (when MCD was used to induce MASH first and anti-ACBP/DBI antibodies were injected later) [27]. Of note, the anti-MASH effects against MCD could be recapitulated using various methods of ACBP/DBI inhibition including whole-body ACBP/DBI knockout and GABRG2 mutation, supporting the idea that the anti-ACBP/DBI antibodies were acting on target [23]. Moreover, in the MCD model, the anti-MASH effects of ACBP/DBI neutralization were lost upon pharmacological inhibition of autophagy with repeated chloroquine injections or upon knockout of *Atg4b*, which is essential for optimal autophagic flux in hepatocytes [23].

Based on these findings, ACBP/DBI inhibition was evaluated in multiple different liver pathologies (Table 3). These included two surgical methods to perturb the integrity and function of the liver, namely transient ligation of the hepatic artery to induce ischemia/reperfusion damage and permanent bile duct ligation to cause cholestasis. In both cases, monoclonal antibodies neutralizing ACBP/DBI reduced the histological signs of necrosis in the liver, as well as the release of transaminases (ALT and AST) from the liver into the circulation. This effect was obtained with a tool antibody recognizing mouse (but not human) ACBP/DBI, as well as in the case for bile duct ligation for an interspecies-specific antibody (that recognizes both human and mouse ACBP/DBI [23, 28]).

**Table 3:** Liver diseases attenuated by ACBP/DBI neutralization.

Disease category	Principle of induction	Disease	Reference
Non-malignant	Transient ligation of hepatic artery	Ischemia-reperfusion damage with liver necrosis	[23]
	Permanent bile duct ligation	Cholestasis-induced liver damage	[23, 28]
	Dietary	High-fat diet-induced steatohepatitis coupled to general obesity	[24]
		Methionine/choline-deficient diet-induced steatohepatitis with body weight loss	[23, 27]
	Drug-induced	Rosiglitazone-induced steatosis	[46]
		Corticosterone-induced steatosis	[49]
	Toxins	Acetaminophen (ferroptosis inducer) induced liver damage	[23]
		Concanavalin A (T cell activation)-induced fulminant hepatitis	[23]
		CCl_4_ (oxidative damage)-induced liver necrosis	[23]
		CCl_4_ combined with oral ethanol challenge, causing liver fibrosis	[27]
	Diet-toxin combination	High-fat diet plus CCl_4_ causing steatohepatitis with fibrosis	[23, 27]
Malignant	Transplanted tumors	Intrahepatic inoculation of Hep55.1 C cells forming orthotopic HCC	[25]
	Oncogene-induced cancers	Hydrodynamic injection of vectors coding for β-catenin and MYC causing HCC	[25]
	Chemical carcinogenesis	High-fat Western-style diet combined with CCl_4_ leading to the development of HCC	[25]
		High-fat Western-style diet plus diethylnitrosamine inducing HCC	[25]

Monoclonal antibodies blocking ACBP/DBI also prevent steatosis induced by rosiglitazone and corticosterone, which both induce enhanced *Dbi* transcription in hepatocytes, rosiglitazone through an action on peroxisome proliferator-activated receptor gamma (PPAR-γ) [46] and corticosterone through the activation of the glucocorticoid receptor [49]. Long-term administration of corticosterone over 6 weeks to mice causes a Cushing-like syndrome characterized by increased food intake, weight gain, adiposity, lipodystrophy, dyslipidemia, insulin resistance, steatosis and a loss of muscle and lean weight (due to sarcopenia and osteoporosis) that can be close-to-fully reversed by all genetic, immunological and pharmacological methods of ACBP/DBI inhibition [49]. Of note, the hepatocyte-specific knockout of *Dbi* is sufficient to curtail the glucocorticoid-induced surge in plasma ACBP/DBI levels (although it does not prevent the high-fat diet-induced elevation in ACBP/DBI) and the manifestation of corticosterone-induced Cushing syndrome, supporting the idea that hepatocytes constitute the major site of ACBP/DBI production relevant to this condition [49]. A plausible scenario is that corticosterone (or other glucocorticoids) has a dual effect on hepatocytes, consisting in the transcriptional activation of the *Dbi* gene, as well as in the induction of autophagic flux, thereby ensuring the secretion of ACBP/DBI into the systemic circulation [52].

A monoclonal antibody specific for mouse ACBP/DBI also protects the murine liver against various toxins including acetaminophen (commercial name: paracetamol), which is a ferroptosis inducer, concanavalin A, which induces fulminant hepatitis due to overactivation of T cells, and carbon tetrachloride (CCl_4_), which causes oxidative necrosis of hepatocytes [23]. Similarly, a dual mouse/human ACBP/DBI-specific antibody shields the liver against acetaminophen-induced hepatotoxicity [28]. Repeated intraperitoneal injections of CCl_4_ cause liver inflammation and fibrosis, especially if combined with a high-fat diet or ethanol, and this pro-fibrotic effect can be prevented or halted by the anti-ACBP/DBI antibody, depending on the timing of administration [23, 27].

In sum, inhibition of ACBP/DBI protects the liver against multiple surgical, dietary, pharmacological and toxicological insults (Table 3). It is important to note that the prevention or reversal of histological signs of liver damage was always accompanied by a reduction of circulating transaminases. Moreover, transcriptomic and metabolomic exploration of the liver confirmed that the hepatoprotective effects of ACBP/DBI inhibition involved a reduction of pro-inflammatory and pro-fibrotic processes as well as the reversal of pathogenic lipid accumulation in the MCD model [23, 27]. Whenever testable, hepatoprotection by ACBP/DBI neutralization depended on the stimulation of autophagic flux [23].

## Prevention and treatment of HCC by targeting ACBP/DBI

Driven by the considerations that (i) metabolic and inflammatory insults of the liver drive hepatocellular carcinogenesis [8, 53], (ii) ACBP/DBI inhibition has broad hepatoprotective and anti-inflammatory effects (Table 3), and (iii) local and circulating ACBP/DBI levels are elevated in HCC patients (Table 1), it has been tempting to explore the possible pathogenic contribution of ACBP/DBI to the initiation and progression of HCC.

As a first approach to tackle the potential role of ACBP/DBI in HCC, short hairpin RNAs (shRNAs) were used to knock down *DBI* or *Dbi* in human and mouse HCC lines, respectively [25]. This manipulation sufficed to strongly reduce the clonogenic survival of the cells in vitro, as well as the proliferation of mouse HCC Hep55.1 C cells in vivo upon their orthotopic inoculation into the livers from immunocompetent, histocompatible mice. These results suggest a cell-autonomous role of ACBP/DBI for optimal HCC growth, yet cannot rule out a potential autocrine or paracrine effect of secreted ACBP/DBI. Importantly, mice in which autoantibodies against ACBP/DBI had been induced by vaccination with an ACBP/DBI-KLH conjugate exhibited a partial reduction of the orthotopic growth of Hep55.1C tumors, pleading in favor of the contribution of extracellular ACBP/DBI to hepatic carcinogenesis [25].

In the next step, hepatocellular oncogenesis was induced by means of the simultaneous hydrodynamic injection of two vectors, one coding for β-catenin and the other for Myc. The combination of these oncogenes usually causes massive hepatocellular oncogenesis within a time frame of 8 weeks. Several manipulations to inhibit the ACBP/DBI system reduced hepatocarcinogenesis, though with different efficacy ranging from close-to-complete protection (upon whole-body conditional knockout of *Dbi*) to more partial effects (upon *Gabrg2*^F77I/F77I^ mutation or induction of ACBP/DBI autoantibodies) [25]. These results can be interpreted to mean that extracellular ACBP/DBI contributes to hepatic oncogenesis, but that only the complete removal of both intra- and extracellular ACBP/DBI confers optimal tumor suppression. Of note, the suppression of the ACBP/DBI system can mediate anticancer effects that occur independently from gross alterations of whole-body metabolism that would be accompanied by shifts in body composition. Indeed, body weight was not affected by the intrahepatic inoculation of cancer cells nor by the administration of oncogenic vectors [25].

The impact of the ACBP/DBI system was also determined in models of MAFLD-associated HCC that was induced by a combination of high-fat Western style chow with extra supplementation of D-glucose and D-fructose in the drinking water combined with one hepatotoxic mutagen, which was either CCl_4_ or diethylnitrosamine (DEN). In both these relatively realistic models of HCC, immunological neutralization of ACBP/DBI by autoantibodies had a clear cancer preventive effect. In addition, in the model of hepatocarcinogenesis driven by Western style diet plus CCl_4_, three different manipulations had marked HCC-preventive effects, namely (i) whole-body knockout of *Dbi*, (ii) hepatocyte-specific knockout of *Dbi* and (iii) the *Gabrg2*^F77I/F77I^ mutation [25]. Again, these findings plead for a pathogenic role of both cancer cell-intrinsic and extracellular ACBP/DBI.

What are then the mechanisms through which the inhibition of ACBP/DBI limits hepatic carcinogenesis? RNAseq of livers undergoing hepatocarcinogenesis by a combination of Western style diet plus CCl_4_ or DEN demonstrated that ACBP/DBI inhibition (by body-wide knockout or autoimmunization) resulted in the consistent upregulation of pro-autophagic and pro-ferroptotic genes, a reduction of cell cycle-relevant mRNAs, as well as a reduction of immunosuppressive gene signatures relevant to regulatory T cells, T helper 2 and T helper 17 cells [25]. Validating these RNAseq data, RT-PCR quantifications of liver mRNAs and immunoblots of liver extracts confirmed the ACBP/DBI-repressed upregulation of multiple pro-ferroptotic genes and proteins, as well as the downregulation of anti-ferroptotic genes and proteins. Moreover, spatially resolved transcriptomics validated the upregulation of ferroptotic and immuno-stimulatory, as well as the downregulation of ferroptosis-inhibitory and immunosuppressive genes by ACBP/DBI knockout or autoantibodies within tumor-free areas of the liver. Immunohistochemical detection of proliferating (Ki67-positive) liver cells revealed an inhibition of the cell cycle advancement both within liver tumors and apparently normal areas of the liver occurring in the context of ACBP/DBI inhibition. Finally, analysis of the liver immune infiltrate by high-dimensional immunofluorescence cytometry confirmed an enhanced intratumoral T cell infiltration, suggestive of improved cancer immunosurveillance [25].

If the aforementioned phenotypic changes had a functional impact, it would be expected that ACBP/DBI neutralization can be advantageously combined with drugs that combat liver cancer by the induction of ferroptosis or by the stimulation of anticancer immune responses. Indeed, injections of an ACBP/DBI-specific monoclonal antibody sensitized mice bearing orthotopic Hep55.1C tumors to the tumor growth-reducing effect of pharmacological ferroptosis inducers such as RSL3 and IKE, as well as to a PD-1 blocking antibody [25].

In sum, neutralization of extracellular ACBP/DBI by autoantibodies or a suitable monoclonal antibody mitigates hepatocellular carcinogenesis in several models. In addition, neutralization of ACBP/DBI sensitizes established HCC to ferroptosis inducers, as well as to PD-1-targeted immunotherapy.

## Conclusions and perspectives

The available evidence suggests that neutralization of ACBP/DBI by suitable monoclonal antibodies has a broad hepatoprotective effect that can be harnessed to prevent and treat a range of non-malignant liver diseases, as well as HCC. That said, there are a few incognita that need to be explored before ACBP/DBI neutralization can be considered as a viable therapeutic avenue.

**Fig. 2:**
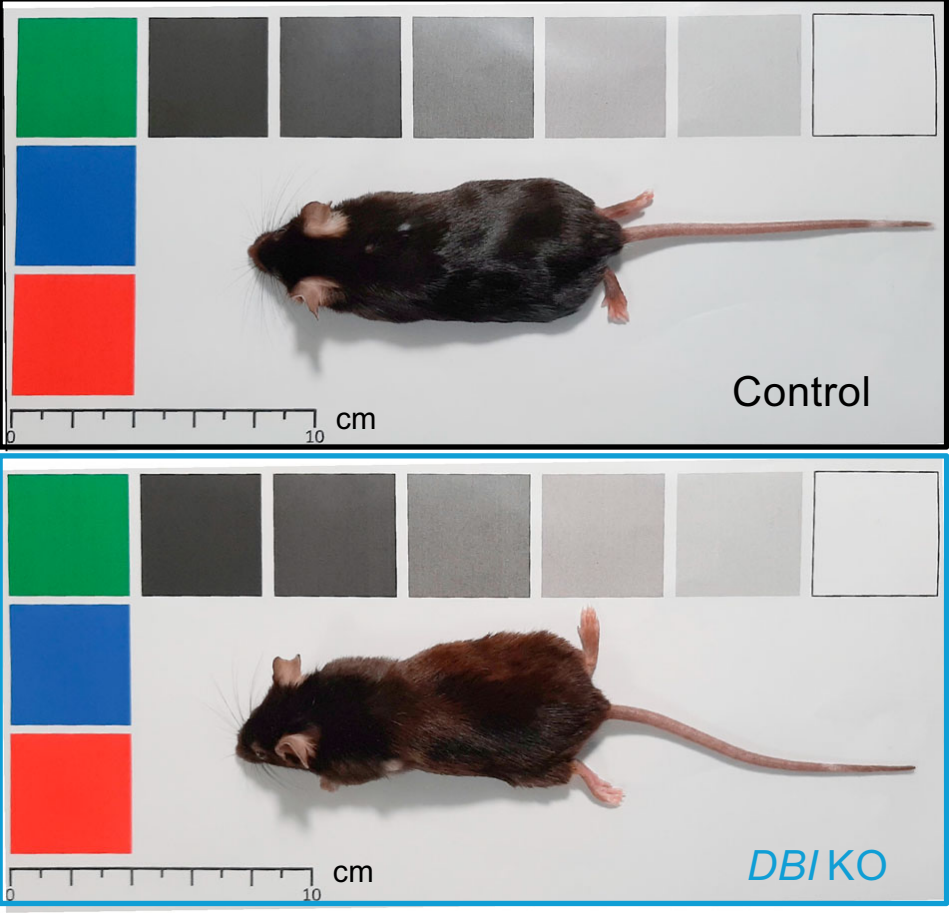
Effect of the whole-body knockout of *DBI* on the macroscopic skin phenotype in C57BL/6J mice in a previously published model of breast cancer induced by medroxyprogesterone acetate and dimethylbenzanthracene [55]. The upper animal is a control mouse (female with the genotype *Acbp/Dbi*^*f*/f^) who received tamoxifen at 5 weeks of age, then followed a tumor-induction protocol starting from 6 weeks of age, and photographed at the age of 8 months. The lower animal is a representative *Dbi* knockout (KO) mouse (female with the genotype *UBC-cre/ERT2*::*Acbp/Dbi*^*f*/f^) which received tamoxifen for Cre activation and followed the same breast tumor induction protocol at the same age. Note that, at endpoint, the *Dbi*^KO^ results in a slightly brownish appearance.

- Dynamic range. The variation of ELISA-detectable ACBP/DBI plasma concentrations among different individuals is relatively small, in the range of 1.5-to-3-fold differences between young healthy control and at-risk or diseased individuals [25–27, 29, 34, 35], suggesting that rather accurate (and perhaps repeated) measurements of ACBP/DBI will be necessary to identify individuals with supraphysiological ACBP/DBI plasma levels that are particularly likely to benefit of ACBP/DBI neutralization. Of course, this caveat only applies if biomarker-based (personalized) interventions were to be incorporated into clinical trials.
- Efficacy. Thus far, the antibody-mediated neutralization of ACBP/DBI has not been able to fully eliminate residual ELISA-detectable ACBP/DBI in the plasma of laboratory mice. Rather, these effects were partial. This may reflect the existence of homeostatic feedback mechanisms (in which free ACBP/DBI is replenished if it is depleted) [5], the underdosing of existing anti-ACBP/DBI antibodies, or the failure of existing ELISAs to accurately distinguish bioavailable from antibody-bound ACBP/DBI. That said, in several disease models, ACBP/DBI-specific antibodies are highly efficient in preventing or treating liver diseases, though sometimes less than the full-body ACBP/DBI knockout.
- Side effects. Thus far, the only side effect of mice subjected to the inducible *DBI* knockout at the adult stage a slight hypopigmentation of the skin and fur, manifesting more than 3 months after whole-body knockout induction (Fig. 2), likely reflecting enhanced autophagy in keratinocytes, which typically causes the removal of melanosomes [54]. A part from this cosmetic effect, no undesirable effects of ACBP/DBI inhibition have been detected thus far, suggesting that such an inhibition is safe. As a caveat, it appears that compensatory hepatocyte proliferation induced by partial hepatectomy in mice is inhibited by ACBP/DBI neutralization [25]. Thus, in particular circumstances, in which such a proliferation may be considered as adaptive (such as in the postoperative phase after removal of large parts of the liver), it may be necessary to avoid the administration of ACBP/DBI-neutralizing antibodies.
- Human trials. Thus far, the entire concept of neutralizing ACBP/DBI for the treatment of liver diseases is based on preclinical evidence obtained in mice and clinical correlations obtained in patient populations. However, to validate this concept, it will be necessary to perform carefully designed clinical trials. We anticipate that the development of interspecies cross-reactive antibodies recognizing ACBP/DBI from humans, rodents and non-human primates will greatly facilitate clinical development because the same antibody (with similar variable but different constant portions) can be characterized in different species.

## Data Availability

Bulk-seq data associated with this study are available in the UCSC Xena platform (https://xenabrowser.net/datapages/).

## References

[1] Dukewich M, Yuan L, Terrault NA. At the crossroads of health and disease: consequences of fat in the liver. Annu Rev Physiol. 2025;87:325–52. 39928502 10.1146/annurev-physiol-022724-105515 PMC12758496

[2] Martini T, Naef F, Tchorz JS. Spatiotemporal metabolic liver zonation and consequences on pathophysiology. Annu Rev Pathol. 2023;18:439–66. 36693201 10.1146/annurev-pathmechdis-031521-024831

[3] Kawashima K, Andreata F, Beccaria CG, Iannacone M. Priming and maintenance of adaptive immunity in the liver. Annu Rev Immunol. 2024;42:375–99. 38360545 10.1146/annurev-immunol-090122-041354

[4] Sawada K, Chung H, Softic S, Moreno-Fernandez ME, Divanovic S. The bidirectional immune crosstalk in metabolic dysfunction-associated steatotic liver disease. Cell Metab. 2023;35:1852–71. 37939656 10.1016/j.cmet.2023.10.009 PMC10680147

[5] Lopez-Otin C, Kroemer G. Hallmarks of health. Cell. 2021;184:33–63. 33340459 10.1016/j.cell.2020.11.034

[6] Rinella ME, Lazarus JV, Ratziu V, Francque SM, Sanyal AJ, Kanwal F, et al. A multisociety Delphi consensus statement on new fatty liver disease nomenclature. J Hepatol. 2023;79:1542–56. 37364790 10.1016/j.jhep.2023.06.003

[7] Sharma S, Le Guillou D, Chen JY. Cellular stress in the pathogenesis of nonalcoholic steatohepatitis and liver fibrosis. Nat Rev Gastroenterol Hepatol. 2023;20:662–78. 37679454 10.1038/s41575-023-00832-w

[8] Karin M, Kim JY. MASH as an emerging cause of hepatocellular carcinoma: current knowledge and future perspectives. Mol Oncol. 2025;19:275–94. 38874196 10.1002/1878-0261.13685 PMC11793012

[9] Singal AG, Kanwal F, Llovet JM. Global trends in hepatocellular carcinoma epidemiology: implications for screening, prevention and therapy. Nat Rev Clin Oncol. 2023;20:864–84. 37884736 10.1038/s41571-023-00825-3

[10] Mizushima N, Levine B. Autophagy in human diseases. N Engl J Med. 2020;383:1564–76. 33053285 10.1056/NEJMra2022774

[11] Rybstein MD, Bravo-San Pedro JM, Kroemer G, Galluzzi L. The autophagic network and cancer. Nat Cell Biol. 2018;20:243–51. 29476153 10.1038/s41556-018-0042-2

[12] Yang X, Cao X, Zhu Q. p62/SQSTM1 in cancer: phenomena, mechanisms, and regulation in DNA damage repair. Cancer Metastasis Rev. 2025;44:33. 39954143 10.1007/s10555-025-10250-w PMC11829845

[13] Takamura A, Komatsu M, Hara T, Sakamoto A, Kishi C, Waguri S, et al. Autophagy-deficient mice develop multiple liver tumors. Genes Dev. 2011;25:795–800. 21498569 10.1101/gad.2016211 PMC3078705

[14] Alquier T, Christian-Hinman CA, Alfonso J. Faergeman NJ. From benzodiazepines to fatty acids and beyond: revisiting the role of ACBP/DBI. Trends Endocrinol Metab. 2021;32:890–903. 34565656 10.1016/j.tem.2021.08.009 PMC8785413

[15] Li S, Mingoia S, Montégut L, Lambertucci F, Chen H, Dong Y, et al. Atlas of expression of acyl CoA binding protein/diazepam binding inhibitor (ACBP/DBI) in human and mouse. Cell Death Dis. 2025;16:134. 40011442 10.1038/s41419-025-07447-w PMC11865319

[16] Montegut L, Abdellatif M, Motino O, Madeo F, Martins I, Quesada V, et al. Acyl coenzyme A binding protein (ACBP): an aging- and disease-relevant “autophagy checkpoint. Aging Cell. 2023;22:e13910. 37357988 10.1111/acel.13910 PMC10497816

[17] Tonon MC, Vaudry H, Chuquet J, Guillebaud F, Fan J, Masmoudi-Kouki O, et al. Endozepines and their receptors: structure, functions and pathophysiological significance. Pharmacol Ther. 2020;208:107386. 31283949 10.1016/j.pharmthera.2019.06.008

[18] Bouyakdan K, Taib B, Budry L, Zhao S, Rodaros D, Neess D, et al. A novel role for central ACBP/DBI as a regulator of long-chain fatty acid metabolism in astrocytes. J Neurochem. 2015;133:253–65. 25598214 10.1111/jnc.13035

[19] Duman C, Yaqubi K, Hoffmann A, Acikgoz AA, Korshunov A, Bendszus M, et al. Acyl-CoA-binding protein drives glioblastoma tumorigenesis by sustaining fatty acid oxidation. Cell Metab. 2019;30:274–89.e275. 31056285 10.1016/j.cmet.2019.04.004

[20] Teng H, Hang Q, Zheng C, Yan Y, Liu S, Zhao Y, et al. In vivo CRISPR activation screen identifies acyl-CoA-binding protein as a driver of bone metastasis. Sci Transl Med. 2025;17:eado7225. 40397713 10.1126/scitranslmed.ado7225 PMC12697304

[21] Blasco-Roset A, Quesada-Lopez T, Mestres-Arenas A, Villarroya J, Godoy-Nieto FJ, Cereijo R, et al. Acyl CoA-binding protein in brown adipose tissue acts as a negative regulator of adaptive thermogenesis. Mol Metab. 2025;96:102153. 10.1016/j.molmet.2025.102153 PMC12050000 40220929

[22] Wu T, Huang T, Ren H, Shen C, Qian J, Fu X, et al. Metabolic coordination structures contribute to diabetic myocardial dysfunction. Circ Res. 2025;136:946–67. 10.1161/CIRCRESAHA.124.326044 40190276

[23] Motino O, Lambertucci F, Anagnostopoulos G, Li S, Nah J, Castoldi F, et al. ACBP/DBI protein neutralization confers autophagy-dependent organ protection through inhibition of cell loss, inflammation, and fibrosis. Proc Natl Acad Sci USA. 2022;119:e2207344119. 36191214 10.1073/pnas.2207344119 PMC9565466

[24] Bravo-San Pedro JM, Sica V, Martins I, Pol J, Loos F, Maiuri MC, et al. Acyl-CoA-Binding Protein is a lipogenic factor that triggers food intake and obesity. Cell Metab. 2019;30:754–67.e759. 31422903 10.1016/j.cmet.2019.07.010

[25] Li S, Motiño O, Lambertucci F, Pol J, Chen H, Pan L, et al. Neutralization of acyl coenzyme A binding protein for the experimental prevention and treatment of hepatocellular carcinoma. Cell Rep Med. 2025;6:102232. 40628264 10.1016/j.xcrm.2025.102232 PMC12281431

[26] Montegut L, Liu P, Zhao L, Perez-Lanzon M, Chen H, Mao M, et al. Acyl-coenzyme a binding protein (ACBP)—a risk factor for cancer diagnosis and an inhibitor of immunosurveillance. Mol Cancer. 2024;23:187. 39242519 10.1186/s12943-024-02098-5 PMC11378439

[27] Motino O, Lambertucci F, Joseph A, Durand S, Anagnostopoulos G, Li S, et al. ACBP/DBI neutralization for the experimental treatment of fatty liver disease. Cell Death Differ. 2025;32:434–46. 39550516 10.1038/s41418-024-01410-6 PMC11894144

[28] Nogueira-Recalde U, Lambertucci F, Montegut L, Motino O, Chen H, Lachkar S, et al. Neutralization of acyl CoA binding protein (ACBP) for the experimental treatment of osteoarthritis. Cell Death Differ. 2025;32:1484–98. 40082721 10.1038/s41418-025-01474-y PMC12326017

[29] Montegut L, Joseph A, Chen H, Abdellatif M, Ruckenstuhl C, Motino O, et al. High plasma concentrations of acyl-coenzyme A binding protein (ACBP) predispose to cardiovascular disease: evidence for a phylogenetically conserved proaging function of ACBP. Aging Cell. 2023;22:e13751. 36510662 10.1111/acel.13751 PMC9835587

[30] Kroemer G, Maier AB, Cuervo AM, Gladyshev VN, Ferrucci L, Gorbunova V, et al. From geroscience to precision geromedicine: understanding and managing aging. Cell. 2025;188:2043–62. 40250404 10.1016/j.cell.2025.03.011 PMC12037106

[31] Eldjarn GH, Ferkingstad E, Lund SH, Helgason H, Magnusson OT, Gunnarsdottir K, et al. Large-scale plasma proteomics comparisons through genetics and disease associations. Nature. 2023;622:348–58. 37794188 10.1038/s41586-023-06563-x PMC10567571

[32] Ferkingstad E, Sulem P, Atlason BA, Sveinbjornsson G, Magnusson MI, Styrmisdottir EL, et al. Large-scale integration of the plasma proteome with genetics and disease. Nat Genet. 2021;53:1712–21. 34857953 10.1038/s41588-021-00978-w

[33] Isnard S, Mabanga T, Royston L, Berini CA, Bu S, Aiyana O, et al. Extracellular acyl-CoA-binding protein as an independent biomarker of COVID-19 disease severity. Front Immunol. 2024;15:1505752. 39835130 10.3389/fimmu.2024.1505752 PMC11743960

[34] Joseph A, Moriceau S, Sica V, Anagnostopoulos G, Pol J, Martins I, et al. Metabolic and psychiatric effects of acyl coenzyme A binding protein (ACBP)/diazepam binding inhibitor (DBI). Cell Death Dis. 2020;11:502. 32632162 10.1038/s41419-020-2716-5 PMC7338362

[35] Joseph A, Chen H, Anagnostopoulos G, Montegut L, Lafarge A, Motino O, et al. Effects of acyl-coenzyme A binding protein (ACBP)/diazepam-binding inhibitor (DBI) on body mass index. Cell Death Dis. 2021;12:599. 34108446 10.1038/s41419-021-03864-9 PMC8190068

[36] Angulo P, Hui JM, Marchesini G, Bugianesi E, George J, Farrell GC, et al. The NAFLD fibrosis score: a noninvasive system that identifies liver fibrosis in patients with NAFLD. Hepatology. 2007;45:846–54. 17393509 10.1002/hep.21496

[37] Sterling RK, Lissen E, Clumeck N, Sola R, Correa MC, Montaner J, et al. Development of a simple noninvasive index to predict significant fibrosis in patients with HIV/HCV coinfection. Hepatology. 2006;43:1317–25. 16729309 10.1002/hep.21178

[38] Sveinbjornsson G, Ulfarsson MO, Thorolfsdottir RB, Jonsson BA, Einarsson E, Gunnlaugsson G, et al. Multiomics study of nonalcoholic fatty liver disease. Nat Genet. 2022;54:1652–63. 36280732 10.1038/s41588-022-01199-5 PMC9649432

[39] De Nardo W, Lee O, Johari Y, Bayliss J, Pensa M, Miotto PM, et al. Integrated liver-secreted and plasma proteomics identify a predictive model that stratifies MASH. Cell Rep Med. 2025;6:102085. 40250425 10.1016/j.xcrm.2025.102085 PMC12147855

[40] Kao CH, Sun LM, Su KP, Chang SN, Sung FC, Muo CH, et al. Benzodiazepine use possibly increases cancer risk: a population-based retrospective cohort study in Taiwan. J Clin Psychiatry. 2012;73:e555–60. 22579162 10.4088/JCP.11m07333

[41] Pottegard A, Friis S, Andersen M, Hallas J. Use of benzodiazepines or benzodiazepine related drugs and the risk of cancer: a population-based case-control study. Br J Clin Pharm. 2013;75:1356–64. 10.1111/bcp.12001 PMC3635606 23043261

[42] Thygesen LC, Pottegard A, Ersboll AK, Friis S, Sturmer T, Hallas J. External adjustment of unmeasured confounders in a case-control study of benzodiazepine use and cancer risk. Br J Clin Pharm. 2017;83:2517–27. 10.1111/bcp.13342 PMC5651330 28599067

[43] Kim HB, Myung SK, Park YC, Park B. Use of benzodiazepine and risk of cancer: a meta-analysis of observational studies. Int J Cancer. 2017;140:513–25. 27667780 10.1002/ijc.30443

[44] Boncompain G, Divoux S, Gareil N, de Forges H, Lescure A, Latreche L, et al. Synchronization of secretory protein traffic in populations of cells. Nat Methods. 2012;9:493–8. 22406856 10.1038/nmeth.1928

[45] Chen H, Moriceau S, Joseph A, Mailliet F, Li S, Tolle V, et al. Acyl-CoA binding protein for the experimental treatment of anorexia. Sci Transl Med. 2024;16:eadl0715. 39141698 10.1126/scitranslmed.adl0715

[46] Anagnostopoulos G, Motino O, Li S, Carbonnier V, Chen H, Sica V, et al. An obesogenic feedforward loop involving PPARgamma, acyl-CoA binding protein and GABA(A) receptor. Cell Death Dis. 2022;13:356. 35436993 10.1038/s41419-022-04834-5 PMC9016078

[47] Landrock D, Atshaves BP, McIntosh AL, Landrock KK, Schroeder F, Kier AB. Acyl-CoA binding protein gene ablation induces pre-implantation embryonic lethality in mice. Lipids. 2010;45:567–80. 20559753 10.1007/s11745-010-3437-9 PMC2997683

[48] Neess D, Bek S, Bloksgaard M, Marcher AB, Faergeman NJ, Mandrup S. Delayed hepatic adaptation to weaning in ACBP-/- mice is caused by disruption of the epidermal barrier. Cell Rep. 2013;5:1403–12. 24316079 10.1016/j.celrep.2013.11.010

[49] Pan H, Tian AL, Chen H, Xia Y, Sauvat A, Moriceau S, et al. Pathogenic role of acyl coenzyme A binding protein (ACBP) in Cushing’s syndrome. Nat Metab. 2024;6:2281–99. 39578649 10.1038/s42255-024-01170-0 PMC11659162

[50] Montegut L, Chen H, Bravo-San Pedro JM, Motino O, Martins I, Kroemer G. Immunization of mice with the self-peptide ACBP coupled to keyhole limpet hemocyanin. STAR Protoc. 2022;3:101095. 35059656 10.1016/j.xpro.2021.101095 PMC8760546

[51] Anagnostopoulos G, Saavedra E, Lambertucci F, Motino O, Dimitrov J, Roiz-Valle D, et al. Inhibition of acyl-CoA binding protein (ACBP) by means of a GABA(A)Rgamma2-derived peptide. Cell Death Dis. 2024;15:249. 38582872 10.1038/s41419-024-06633-6 PMC10998878

[52] Pan H, Tian AL, Castinetti F, Martins I, Kepp O, Kroemer G. Autophagy-dependent hepatocyte secretion of DBI/ACBP induced by glucocorticoids determines the pathogenesis of Cushing syndrome. Autophagy. 2025;21:678–80. 39663572 10.1080/15548627.2024.2437649 PMC11849933

[53] Font-Burgada J, Sun B, Karin M. Obesity and cancer: the oil that feeds the flame. Cell Metab. 2016;23:48–62. 26771116 10.1016/j.cmet.2015.12.015

[54] Lee KW, Kim M, Lee SH, Kim KD. The function of autophagy as a regulator of melanin homeostasis. Cells 2022;11:2085. 10.3390/cells11132085 PMC9265842 35805169

[55] Buque A, Bloy N, Perez-Lanzon M, Iribarren K, Humeau J, Pol JG, et al. Immunoprophylactic and immunotherapeutic control of hormone receptor-positive breast cancer. Nat Commun. 2020;11:3819. 32732875 10.1038/s41467-020-17644-0 PMC7393498

[56] Montegut L, Derosa L, Messaoudene M, Chen H, Lambertucci F, Routy B, et al. Benzodiazepines compromise the outcome of cancer immunotherapy. Oncoimmunology. 2024;13:2413719. 39381589 10.1080/2162402X.2024.2413719 PMC11459736

